# Study of the Resistance of *Staphylococcus aureus* Biofilm, Biofilm-Detached Cells, and Planktonic Cells to Microencapsulated Carvacrol Used Alone or Combined with Low-pH Treatment

**DOI:** 10.3390/ijms25137222

**Published:** 2024-06-29

**Authors:** Samah Mechmechani, Jina Yammine, Sakhr Alhuthali, Majededdine EL Mouzawak, Georgia Charvourou, Adem Ghasrsallaoui, Nour Eddine Chihib, Agapi Doulgeraki, Layal Karam

**Affiliations:** 1 Institut National de Recherche Pour L’agriculture, L’alimentation Et L’environnement (INRAE), University of Lille, Centre national de la recherche scientifique (CNRS), 59120 Lille, France; samah.mechmchani108@gmail.com (S.M.); jina.yammine@gmail.com (J.Y.); nour-eddine.chihib@univ-lille.fr (N.E.C.); 2 Department of Chemical and Materials Engineering, Faculty of Engineering, King Abdulaziz University, Jeddah 22233, Saudi Arabia; 3 Department of Chemical Engineering, Imperial College London, London SW7 2AZ, UK; 4 Institut Lillois d’Ingénierie de la Santé, (ILIS), 59120 Lille, France; majdeddine.mzawak@outlook.com; 5 Institute of Technology of Agricultural Products-Hellenic Agricultural Organization DIMITRA, S. Venizelou 1, 14123 Lycovrissi, Greece; georgia.charvourou@gmail.com; 6 Laboratoire d’Automatique, de Génie des Procédés et de Génie Pharmaceutique, CNRS, University Claude Bernard Lyon 1, 43 Bd 11 Novembre 1918, 69622 Villeurbanne, France; adem.gharsallaoui@univ-lyon1.fr; 7 Laboratory of Food Microbiology and Hygiene, Department of Food Science and Technology, Faculty of Agriculture, Forestry and Natural Environment, School of Agriculture, Aristotle University of Thessaloniki, 54124 Thessaloniki, Greece; 8 Human Nutrition Department, College of Health Sciences, QU Health, Qatar University, Doha P.O. Box 2713, Qatar

**Keywords:** biofilm, biofilm-detached cells, planktonic cells, microencapsulated carvacrol, *Staphylococcus aureus*

## Abstract

Microbial biofilms pose severe problems in the medical field and food industry, as they are the cause of many serious infections and food-borne diseases. The extreme biofilms’ resistance to conventional anti-microbial treatments presents a major challenge to their elimination. In this study, the difference in resistance between *Staphylococcus aureus* DSMZ 12463 biofilms, biofilm-detached cells, and planktonic cells against microcapsules containing carvacrol was assessed. The antimicrobial/antibiofilm activity of low pH disinfection medium containing the microencapsulated carvacrol was also studied. In addition, the effect of low pH on the in vitro carvacrol release from microcapsules was investigated. The minimum inhibitory concentration of microencapsulated carvacrol was 0.625 mg mL^−1^. The results showed that biofilms exhibited greater resistance to microencapsulated carvacrol than the biofilm-detached cells and planktonic cells. Low pH treatment alone, by hydrochloric acid addition, showed no bactericidal effect on any of the three states of *S. aureus* strain. However, microencapsulated carvacrol was able to significantly reduce the planktonic cells and biofilm-detached cells below the detection limit (no bacterial counts), and the biofilm by approximatively 3 log CFU mL^−1^. In addition, results showed that microencapsulated carvacrol combined with low pH treatment reduced biofilm by more than 5 log CFU mL^−1^. Thus, the use of microencapsulated carvacrol in acidic environment could be a promising approach to combat biofilms from abiotic surfaces.

## 1. Introduction

*Staphylococcus aureus* is a serious food poisoning pathogen, implicated in a broad range of diseases related to soft tissue and benign skin infections, and illnesses such as osteomyelitis and endocarditis [1]. The worldwide spread of methicillin-resistant *S. aureus* (MRSA) has become one of the world’s most pressing public health concerns. This microorganism has developed new resistance mechanisms to modern antibiotics and is a common cause of infections linked to biofilms associated with the widespread use of medical devices [2,3] and in the food industry [4].

Biofilms are currently regarded as one of the most significant bacterial virulence factors, as they substantially contribute to microbial survival in harsh environments. Biofilm formation is known as a major driver of antibiotic resistance and the pervasiveness of *Staphylococcus aureus* infections [5]. They are widely recognized for their greater resistance to antimicrobial agents and other environmental stressors, compared to their planktonic (free-floating) counterparts, making them very difficult to remove [6]. Moreover, biofilms serve as a reservoir of bacteria, which can pose a significant threat when cells detach from the biofilm structure. These detached cells represent sources of contamination for medical and food contact surfaces, potentially leading to serious diseases. Once these cells leave the biofilm environment, they often exhibit a higher level of resistance, rendering them a real menace that requires efficient antimicrobial treatment [7]. In general, bacterial biofilms confer dual features to microbial cells, enhanced resistance to antibiotics, as well as increased invasiveness capabilities [8]. For this reason, the development of alternative strategies or more potent antimicrobial agents capable of effectively targeting microorganisms that produce biofilms is of great interest and importance.

Bio-based antimicrobial agents have gained significant attention in the recent years due to their high efficiency, safety, and non-toxic properties. Essential oils, i.e., aromatic liquids extracted from plant matter, are considered natural compounds that are biodegradable, eco-friendly, and non-toxic, yet possess potent antimicrobial capabilities to combat various types of contamination [9]. Many studies have demonstrated the ability of essential oils to effectively eliminate and prevent the formation of biofilms [10,11,12,13,14]. To date, the majority of the published studies in the literature have not reported any evidence of specific antimicrobial resistance developed towards essential oils [15]. This is attributed to the fact that essential oils exhibit multiple mechanisms of action, targeting various bacterial cell sites simultaneously [16,17]. A limited number of studies have demonstrated the varying sensitivity of bacterial isolates against essential oils, and the subsequent development of resistance to antimicrobials [18,19].

However, more research is needed to provide solid evidence that different bacterial strains may develop resistance against essential oils. The monoterpene phenol carvacrol [2-Methyl-5-(1-methylethyl) phenol, isomer of thymol] is a main constituent of the essential oils of the plants of the Labiatae family, including *Thymus* and *Origanum*, which have been widely used as condiments and in folk medicine from ancient times [20]. Carvacrol has been graded as GRAS (Generally Recognized As Safe) and certified for application in food [21,22]. In addition to its antioxidant, anti-inflammatory, analgesic, antitumor, insecticidal and antihepatotoxic properties, numerous studies have demonstrated that carvacrol also exhibits potent antimicrobial activities. Furthermore, several other studies have assessed the antibiofilm properties of carvacrol on commonly used food and medical surfaces [20,23,24,25].

Nevertheless, the application of essential oils is restricted by their inherent low stability, high volatility, and poor water solubility [26]. To overcome these limitations, a novel strategy based on the encapsulation of these molecules within various material carriers is needed. Microencapsulation of essential oils is an effective approach to improve their stability and reduce their immiscibility in water [27]. Additionally, this method allows for the controlled release of the active compounds and minimizes their physicochemical interactions with the biofilm matrix components. These interactions are often linked to a reduction in the effectiveness of anti-biofilm molecules [12,27,28]. Another approach adopted for the successful application of essential oils is to combine them with other treatments. Frequently, combination of essential oils with other antimicrobial interventions has resulted in more potent antimicrobial activity. This strategy also enables the use of reduced quantities of essential oils, thereby minimizing environmental and sensory impacts, lowering production costs, and reducing the associated risk of antimicrobial resistance emergence [29,30,31,32]. Several researchers have shown the enhanced activity of essential oils in inactivating biofilms when combined with other components such as enzymes [33], antibiotics [13], and cold nitrogen plasma [28]. However, a gap remains in research on combining essential oils with acidic treatments. Acidic disinfectants can involve organic compounds (e.g., citric acid, acetic acid) as well as inorganic compounds (e.g., sulfuric acid, hydrochloric acid). The antimicrobial activity of acids occurs through the dissociation of free hydrogen ions, which alters the pH of the microorganism’s environment. Acids can also precipitate proteins and destroy nucleic acid bonds. Furthermore, it has been hypothesized that altering the pH modifies the surface charge, which can prevent bacterial attachment, an essential step in biofilm formation [34].

In this study, we examined the effect of microencapsulated carvacrol, alone or combined with a low-pH treatment using hydrochloric acid (HCl), on three different states of the *S. aureus* strain: planktonic cells, biofilm, and biofilm-detached cells. The main objectives were to demonstrate the differences in resistance to antimicrobial treatment between these three bacterial states, and to explore whether the antimicrobial activity of microencapsulated carvacrol could be enhanced by applying a low-pH condition. By investigating the efficacy of this combinatorial approach, the study aims to provide insights into improving the effectiveness of essential oil-based antimicrobial strategies against the various forms of *S. aureus*.

## 2. Results

### 2.1. Carvacrol Dropels Zeta Potantial

During the preparation of the carvacrol microcapsules, the emulsion’s pH was adjusted to 7. The zeta potential measurement of the dispersed droplets yielded an average value of −24.39 mV. This negative value is attributed to the use of sodium caseinate as an emulsifier at a pH higher than its isoelectric point (pHi~4.5). When the pH exceeds the pHi, proteins tend to exhibit an overall negative charge due to the ionization of carboxyl groups (COOH^−^ > COO^−^).

To investigate the impact of the spray-drying process, which involves thermomechanical treatment combining shearing and heating, the zeta potential was measured after reconstitution of the powder. The average value obtained was −23.57 mV, indicating that the presence of maltodextrins as a drying matrix helps maintain the structure of the protein interfacial membrane surrounding the carvacrol droplets.

### 2.2. SEM Structure of Dried Microcapsules

**Figure 1:**
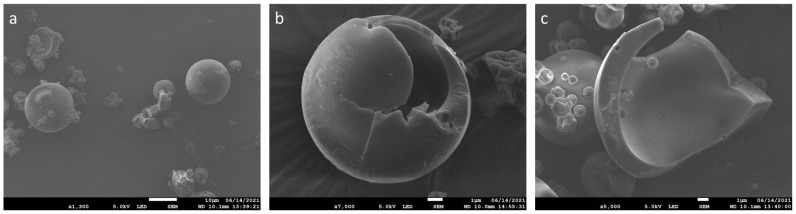
SEM images of carvacrol spray-dried microcapsules: (**a**) outer structure of carvacrol microcapsules (1300×); (**b**,**c**) inner structure of carvacrol microcapsules (7000× and 5000×).

Figure 1 illustrates scanning electron micrography (SEM) of microcapsules produced by spray-drying emulsions containing carvacrol alongside maltodextrins DE19 and sodium caseinate. The microcapsules are depicted as spherical and well-separated, exhibiting non-uniform diameters. They display a blunted shape and generally bumpy surfaces, with the presence of some small, shrunken particles characterized by a rough surface (Figure 1a). Internal structure observations reveal the existence of a central boundary air bubble, termed a void (Figure 1b). Furthermore, the wall matrix of these microcapsules appears thickened and typically hollow, with an evident encapsulated core material retained within (Figure 1c).

### 2.3. Minimal Inhibitory Concentration of Microencapsulated Carvacrol

The antibacterial activity of microencapsulated carvacrol was evaluated against *S. aureus* strain DSMZ 12463 using a microdilution growth inhibition assay. Results showed that the lowest concentration of microencapsulated carvacrol that inhibits *S. aureus* strain growth was 0.625 mg mL^−1^.

### 2.4. Carvacrol In Vitro Release Analysis

**Figure 2:**
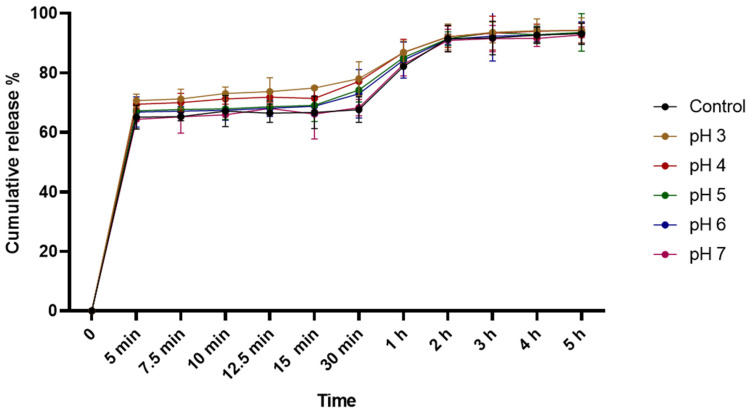
Cumulative in vitro release profile of carvacrol from microcapsules using phosphate buffered saline (PBS, pH 7.0) at varying pH values. Results are expressed as mean ± SD.

The in vitro release profile of carvacrol was investigated to understand the effect of different pH values on the release of carvacrol from the microcapsules. The release profiles were monitored in a phosphate buffered saline (PBS) solution at pH 7.0. As shown in Figure 2, the release of carvacrol from the microcapsules was characterized by a rapid initial release within the first 5 min, with 65.08% of the carvacrol released for the control solution. This initial release phase reached 66.69% after 15 min (the antimicrobial treatment time) and lasted around 2 h, with 91.52% of the carvacrol released. Thereafter, the carvacrol release rates slowed and achieved a stable state, with the highest cumulative release of 93.16% after 5 h.

The results also showed no significant difference in the carvacrol release profile of the microcapsules between the control and the different pH values tested (*p* > 0.05). Even at the lowest pH of 3, the initial rapid release during the first 5 min reached 70%. This release rate reached 74.89% after 15 min and then slowed down, ultimately reaching 94.18% after 5 h of resuspension in the PBS solution. The same release profile was observed for the other solutions with different pH values.

### 2.5. Antimicrobial Activity of Microencapsulated Carvacrol against Planktonic Cells, Biofilm, and Biofilm-Detached Cells of Staphylococcus aureus

The assessment of the antimicrobial activity of microencapsulated carvacrol and/or low-pH treatment was performed on three bacterial states of *S. aureus*: planktonic cells, biofilm-detached cells, and biofilm. Results showed that after 24 h of incubation, the control cell suspensions exhibited bacterial counts of 5.7 CFU mL^−1^ for planktonic cells, 4.9 CFU mL^−1^ for biofilm-detached cells, and 5.3 CFU mL^−1^ for biofilms.

When the bacterial cells were exposed to low-pH treatment using HCl solution at different pH values, there was no significant bactericidal effect on any of the three bacterial states, and thus no notable difference in resistance to the HCl solution among the three bacterial states.

**Figure 3:**
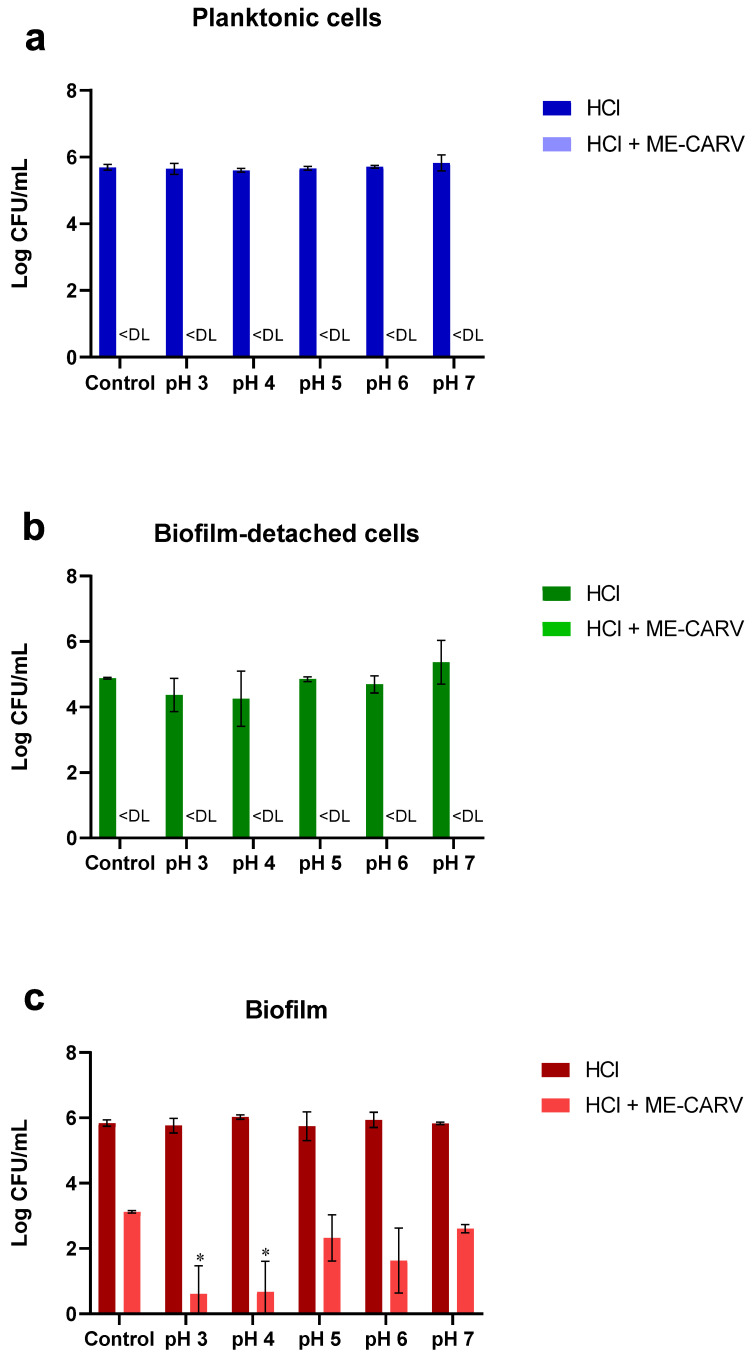
Effect of microencapsulated carvacrol (at MIC concentration: 0.625 mg mL^−1^) and/or low-pH treatment using HCl on the total count of *Staphylococcus aureus* DSMZ 12463 planktonic cells (**a**), biofilm-detached cells (**b**), and biofilm (**c**). Control of HCl treatment represents bacterial strain treated with ¼ Ringer solution. Control of combined treatment represents bacterial strain treated with ¼ Ringer solution containing microencapsulated carvacrol alone. * *p <* 0.05 indicates a significant difference compared with control using Dunnett’s multiple comparisons test. < DL represents cell count below the detection limit.

However, the results showed that treatment using microencapsulated carvacrol at the minimum inhibitory concentration (MIC) was able to significantly reduce the planktonic cells and biofilm-detached cells below the detection limit (no bacterial counts) (*p* < 0.05) (Figure 3a,b), and the biofilms by approximately 3 log CFU mL^−1^ (*p* < 0.05) (Figure 3c).

Importantly, the combination of microencapsulated carvacrol and low pH using HCl was able to reduce the planktonic cells and biofilm-detached cells below the detection limit across all pH ranges. Additionally, this combinatorial treatment reduced the biofilm by more than 5 log CFU mL^−1^ at pH 3 and pH 4 (*p <* 0.05) (Figure 3c). These results demonstrate that the low pH enhanced the antimicrobial activity of carvacrol.

## 3. Discussion

The application of natural plant-based substances, including essential oils, has become increasingly popular as an alternative to synthetic biocides [35]. However, these plant-extracted compounds often face challenges related to their stability, as they are volatile and susceptible to photolysis and oxidation. Additionally, their low solubility can reduce their antimicrobial effectiveness [26,36,37]. In our study, we addressed these issues by employing a microencapsulation technique. The sodium caseinate was selected as an emulsifier. This choice was based on its ability to form a protective membrane around carvacrol droplets, and we further supplemented it with maltodextrins as a wall material to enhance encapsulation efficiency [38].

SEM analysis was employed to examine the microstructure of spray-dried carvacrol microcapsules. Figure 1 illustrates that the microparticles exhibited a spherical shape with bumpy surfaces. The irregular surfaces observed may be attributed to shrinkage during the drying and cooling stages, a characteristic of the spray-drying process [39,40]. Investigation of the internal structure demonstrated that carvacrol microcapsules were hollow, featuring a central void. The presence of air holes in the microcapsule walls indicated the presence of volatile compounds within the capsules. These voids may result from air expansion during the spray-drying process within the droplets [41].

Microencapsulated carvacrol was first evaluated for its antibacterial action before testing its efficacy on biofilms. The MIC of microencapsulated carvacrol against *S. aureus* DSMZ 12463 was 0.625 mg mL^−1^. Microcapsules without carvacrol were used as a negative control and showed no antimicrobial activity against the strain used. In a previous study, microencapsulated carvacrol demonstrated inhibitory activity against an *S. aureus* strain at a lower MIC of 225 ppm (0.225 mg mL^−1^) [42]. Carvacrol is one of the two main constituents, along with thymol, of oregano essential oil. This phenolic compound exhibits remarkable antibacterial activity and is currently being explored as a natural alternative to chemically active agents in the formulation of disinfectants for cleaning equipment [43]. Carvacrol exhibits its antimicrobial activity by impairing membrane integrity, resulting in leakage of cytoplasmic contents such as nucleic acids and lactate dehydrogenase enzymes [44,45]. The hydrophobic properties of this active compound enable it to interfere with the bacterial cytoplasmic membrane’s lipid bilayer. As it aligns across the fatty acid chains, carvacrol disrupts the structure of the membrane bilayer, disrupting its permeability and fluidity, resulting in leakage of internal cellular material [6,20]. However, the use of free carvacrol is restricted due to its water immiscibility, poor stability, and extreme volatility. Therefore, microencapsulation of this natural active compound could be a prominent method to overcome those limitations that reduce the activity and shelf life of the essential oil [6,26]. Previous studies showed the enhanced antibacterial activity of microencapsulated carvacrol compared to their free counterparts against several bacterial strains [6,46]. For example, Mechmechani et al. (2022) showed that the minimum inhibitory concentration (MIC) of encapsulated carvacrol (E-CARV) against *Pseudomonas aeruginosa* was 4 times lower (1.25 mg/mL) compared to free carvacrol (F-CARV) (5 mg/mL). Furthermore, the exposure to E-CAR induced greater membrane destabilization, morphological deformations, and leakage of cellular contents in the bacteria compared to the free antimicrobials [6]. Similarly, Yammine et al. (2023) found that the MIC values for free carvacrol (CAR) and thymol (THY) against *Salmonella enteritidis* were both 1.25 mg/L. However, nanoencapsulation reduced the MIC to 0.62 mg/L for THY and 0.31 mg/L for CAR [46]. The improved antimicrobial activity of the encapsulated essential oils versus several types of microbial contamination can be mainly related to the small particle size and increased surface-to-volume ratio, enabling the diffusion of essential oils in the microbial cells [28,47]. Encapsulation can decrease essential oils’ volatility and degradation, which could be possibly correlated to an increase in their antimicrobial activity [6,48]. Moreover, the diffusion of essential oils towards cell membranes can be facilitated by the encapsulation process, since free essential oils are not very soluble in water and therefore have difficulty interacting with membranes, whereas encapsulated essential oils are more soluble [49].

In addition, the difference in resistance between *S. aureus* biofilms, biofilm-detached cells, and planktonic cells to microencapsulated carvacrol and/or low-pH treatment using HCl was investigated using cell counts. Planktonic cells were prepared in TSB medium, and biofilms were performed on stainless steel slides. This experimental setup was chosen because biofilms and biofilm-detached cells represent a main source of microbial contamination and dissemination in the medical and food sectors [27,50]. Research into the resistance of *S. aureus* biofilms, biofilm-detached cells, and planktonic cells is crucial to better assess the microbiological risks associated with these bacterial states, and to improve the effectiveness of appropriate disinfection procedures. Stainless steel was chosen as the biofilm growth surface because it is a material commonly found in food and medical equipment [51]. Our results showed that low-pH treatment using HCl did not affect the three states of the *S. aureus* strain, and there was no significant reduction in bacterial counts across biofilms, biofilm-detached cells, and planktonic cells. This is in contrast with previous studies that have demonstrated varying antimicrobial activities of different types of acids. For example, Kundukad et al. (2020) found that at pH 2.5, acetic acid treatment could increase the number of dead cells in *S. aureus* biofilms by only 10% (from 60 ± 31% viable cells before treatment to 50 ± 43% viable cells after treatment), whereas N-acetylcysteine acid treatment can increase the percentage of dead cells in *S. aureus* by about 65% [52]. Bjarnsholt et al. (2015) demonstrated that acetic acid exhibited a strong antimicrobial activity against the *S. aureus* strain at pH 4.76 resulting from the undissociated form of acetic acid, whereas the use of HCl at the same pH did not result in any antimicrobial activity [53]. However, Ekpiken et al. (2017) showed that HCl treatment of *S. epidermidis* biofilm at 1 M showed a remarkable global reduction in EPS production compared with the control and thus a significant inhibition of biofilm formation [54]. Another study showed that all *S. aureus* cells in suspension were killed after 10 min of exposure to 0.1 M HCl [55]. Thus, the efficiency of acid treatments is affected by several factors including the type of acid (weak or strong), the concentration used or pH, target microorganism, the growth phase, and the state of the bacteria (planktonic or sessile).

On the other hand, the results showed that treatment with microencapsulated carvacrol was highly effective against the *S. aureus* strain. The microbial count of the planktonic cells and biofilm-detached cells was reduced to below the detection limit, while the biofilm was reduced by approximately 3 log CFU mL^−1^. These results demonstrate the potent antimicrobial activity of microencapsulated carvacrol against this bacterial strain, which is highly significant given that the *S. aureus* pathogen has developed resistance mechanisms to the vast majority of antimicrobial drugs used in treatment, namely the beta-lactam, glycopeptide, and oxazolidinone drugs [56]. These findings align with previous studies that have also reported the strong antimicrobial properties of carvacrol against *S. aureus*. For instance, Hao et al. (2021) explored the antibacterial activity and mechanism of action of carvacrol-rich oregano essential oil (OEO) derived from Origanum vulgare on planktonic *S. aureus* cells. They showed that the carvacrol-rich OEO displayed a strong antibacterial effect, reducing peak bacterial populations, decreasing bacterial viability, and damaging the cell wall [57]. Another study also showed that carvacrol inhibited multiple targets against methicillin-resistant *S. aureus* (MRSA) [58]. Furthermore, Wang et al. (2020) demonstrated that carvacrol significantly inhibited the *S. aureus* biofilm formation by reducing the autoinducer-2 of quorum sensing [59]. Similarly, Walczak et al. (2021) showed that carvacrol was able to reduce the amount of *S. aureus* biofilm by 95–100% after several days’ exposure [60]. Espina et al. (2017) reported that carvacrol treatment was capable of decreasing sessile cells’ formation of the mature *S. aureus* biofilm by more than 5 log cycles [61].

Additionally, our results showed that there was no difference in resistance between *S. aureus* planktonic and biofilm-detached cells at a 0.625 mg mL^−1^ concentration of microencapsulated carvacrol. Similarly, Rollet et al. (2009) reported that planktonic, biofilm-detached, and sessile *P. aeruginosa* cells show the same sensitivity profile to antibiotics [62]. Contrarily, Bolets et al. (2008) demonstrated that biofilm-detached cells of the *S. aureus* strain exhibited greater resistance to Rifampicin antibiotic than planktonic cells, suggesting that part of the detached biofilm may remain in the form of emboli (aggregated cell masses) that are known to possess elevated antibiotic resistance [7]. However, our results showed that bacterial biofilms exhibit significantly greater resistance compared to planktonic cells and biofilm-detached cells. The same treatment and concentration that were capable of fully eliminating the planktonic and biofilm-detached bacterial states could only achieve a 3-log reduction in the biofilm state. The inability of the treatment to fully eliminate the biofilm state, in contrast to its effectiveness against other bacterial forms, highlights the heightened resistance mechanisms conferred by the biofilm mode of growth. These findings align with many previous studies that have reported bacteria in a biofilm state can display 10 to 1000 times greater antimicrobial resistance than their planktonic counterparts [27,63,64,65,66]. This enhanced antimicrobial resistance of the biofilm arises from several mechanisms that limit diffusion of antimicrobial agents through the biofilm’s extracellular polymer matrix, enzyme-mediated resistance within the biofilm, interactions between the antimicrobial agent and the biofilm matrix (cells and polymers), genetic adaptation of biofilm cells, altered metabolic activity levels within the biofilm, changes to the structure of the outer membrane, and increased efflux pump activity [67,68,69,70,71,72,73,74,75,76].

Our results also showed that the combined treatment of microencapsulated carvacrol and low pH (pH 3 and pH 4) resulted in a significant increase in biofilm reduction of over 5 log CFU mL^−1^ compared to microencapsulated carvacrol alone. This suggests that the low pH enhances the antimicrobial activity of the microencapsulated carvacrol against the biofilm. To investigate the mechanism behind this enhanced antimicrobial effect, the in vitro release profile of carvacrol from the microcapsules was assessed at different pH values over a 5-h period. The results showed that the majority of carvacrol was rapidly released from the microcapsules within the first 5 min of being resuspended in the PBS solution, regardless of the pH. The release rate then slowed down after 2 h, reaching the highest cumulative release by the end of the 5-h period. The rapid initial release of carvacrol from the microcapsules can be attributed to the properties of the carrier polymer used, which was maltodextrin. Maltodextrin is a highly soluble polymer due to the presence of hydroxyl groups within its structure. When the microcapsules were resuspended in the aqueous PBS solution, the hydroxyl groups on the maltodextrin were able to readily interact with the water molecules. This allowed for quick hydration and dissolution of the carrier polymer. As a result, the encapsulated carvacrol was quickly released from the microcapsules once they were suspended in the aqueous medium [77,78,79]. It is also likely that carvacrol molecules close to or adsorbed on the capsule surface were quickly liberated during this early phase, as they had a low affinity for the carrying mate-rial [80]. The late release phase of carvacrol from microcapsules, with a slow and sustained release over several hours, could be largely due to the extended period required for release of carvacrol from the shell coating [81]. Results also showed that pH did not affect the release of carvacrol from microcapsules. That might be due to the high stability of maltodextrin, used as wall material, under acidic conditions, so that diffusion of carvacrol from the microcapsules had the same profile at different pH values [82]. These results suggest that the antimicrobial activity of microencapsulated carvacrol at low pH is not mainly attributed to the increased release of carvacrol from the microcapsules, but to the potential synergy between low pH and microencapsulated carvacrol treatment. A previous study demonstrated that the application of peracetic acid combined with essential oils improved its efficacy against *Listeria monocytogenes* biofilms [83]. In addition, Nostro et al. (2012) demonstrated that the use of sub-inhibitory concentrations (1/2, 1/4, and 1/8 MIC) of carvacrol at acidic pH resulted in a greater reduction in *S. aureus* and *S. epidermidis* biofilms compared to that observed at neutral pH [84]. This increased activity could be linked to the fact that at a low-pH level, carvacrol becomes more dissociated and hydrophobic, leading to greater partitioning into the lipid layer of the bacterial membrane, thereby increasing its damage [85,86]. Furthermore, it can be inferred that acidic pH modifies the ionic interactions of the biofilm’s polar matrix, allowing greater diffusion of carvacrol in this matrix

## 4. Materials and Methods

### 4.1. Bacterial Strain Preparation and Cell Suspension

The target microorganism used in this study was *S. aureus* DSMZ 12463. The strain was kept in Tryptic Soy Broth (TSB; Biokar Diagnostics, Allonne, France) plus glycerol (30% *w*/*w*) (AppliChem, Darmstadt, Germany) at −80 °C. Bacteria were precultured by inoculating 10 μL in 5 mL of TSB and incubated for 24 h at 37 °C. Then 100 μL of the pre-culture was used to inoculate 5 mL of TSB medium and incubated for 16 h at 37 °C for the preparation of the culture. Cells were centrifuged at 5000× *g* for 5 min. Harvested cells were double washed with 20 mL of ¼ Ringer’s solution (Lab M Ltd., Lancashire, UK) and then resuspended in 10 mL of ¼ Ringer’s solution.

### 4.2. Antimicrobial Conponents

Carvacrol was obtained from Sigma-Aldrich (98% purity, St. Louis, MO, USA). The different pH solutions (pH 3, pH 4, pH 5, pH 6, pH 7) were prepared using ¼ Ringer solution and HCl (0.1 or 1 mol L^−1^) or NaOH (0.1 or 1 mol L^−1^).

### 4.3. Carvacrol Microencapsulation

Carvacrol was microencapsulated using the spray-drying method according to Mechmechani et al. (2022) [6]. Briefly, after preparation of the carvacrol emulsions with sodium caseinate, emulsions were stirred with a solution of maltodextrin DE 19 (50% *w*/*v*), resulting in a feed emulsion containing 1% carvacrol, 0.5% sodium caseinate, and 20% maltodextrin. The emulsions were then stirred and spray dried using laboratory equipment equipped with a nozzle atomizer of 0.5 nm (Mini Spray-Dryer Buchi B-290, Flawil, Switzerland). Operating conditions for the drying process were inlet air temperature 180 ± 2 °C, outlet air temperature 80 ± 5 °C, and feed rate 0.5 L h^−1^. After spray drying, powders were gathered in separate containers and stored at 4 °C.

### 4.4. Characterisation of the Carvacrol Microcapsule: Zeta Potantial Measurment and Scaning Electron Microscopy Observation

The ζ-potential, or electrical charge, of the emulsions was measured both before and after spray drying and reconstitution using a Zetasizer Nano ZS90 (Malvern Instruments, Malvern, UK). If necessary, samples were diluted in water with the required pH. Each test was repeated at least three times, and the average ζ-potential (ZP) values were calculated from the instrument readings.

In addition, the external and internal structures of the microcapsules were examined using a scanning electron microscope (JEOL-JSM-7800FLV, Tokyo, Japan). from Japan. For observing the external structure, a dry powder layer was simply attached to a sample holder using double-sided adhesive (Agar Scientific, Stansted, UK). To investigate the internal structure, powders containing carvacrol microcapsules were crushed by passing a razor blade perpendicularly through a layer of microcapsules. Prior to scanning by SEM, the fixed bacterial cells or dried microcapsule samples were coated with a thin carbon film. SEM imaging was conducted at 3 kV.

### 4.5. Determination of the Minimal Inhibitory Concentration of Microencapsulated Carvacrol against Staphylococcus aureus Planktonic Cells

The minimum inhibitory concentration of microencapsulated carvacrol against *S. aureus* strain was determined using Tryptic Soy Agar (TSA) with a growth inhibition microdilution assay via a microplate reader that detects vertical turbidity photometry. Firstly, 100 μL of serial double dilutions of microencapsulated carvacrol (from 10 to 0.156 mg mL^−1^) was made in 96-well microdilution plates. Next, 100 μL of the *S. aureus* strain adjusted to 10^6^ CFU mL^−1^ was added. In the negative control, no bacteria were added, whereas in the positive controls, an antimicrobial-free medium was used. The plate was incubated in a microplate reader at 37 °C with continuous agitation and the optical density (OD 610 nm) was read every 10 min for 24 h. T The MIC value was determined as the lowest antimicrobial agent concentration that impedes obvious bacterial proliferation in the microdilution wells following incubation.

### 4.6. Study of the In Vitro Release of Carvacrol from Microcapsules

The release profile of carvacrol from the microcapsules was performed as described previously by Yammine et al., 2023 [46]. Briefly, microcapsules suspensions were prepared in NaCl solution at 10 mg/mL. Next, the prepared suspensions were placed in a dialysis bag (Thermo Fisher Scientific, Altrincham, UK) with a molecular weight cut-off of 3500 Da, which is permeable to carvacrol (MW < 200 Da) but not to the other constituents of the microcapsules (MW > ≈20 kDa). The dialysis bag was securely sealed and then placed in TSB solutions acidified to different pH conditions (3.0, 4.0, 5.0, 6.0, and 7.0), while gently stirring at room temperature. The release tests of carvacrol at different pH conditions were conducted at specific time intervals (5 min, 7.5 min, 10 min, 12.5 min, 15 min, 30 min, and 1 to 5 h, by measuring the release every one hour). This was performed by withdrawing 3 mL samples from the outside of the dialysis bags and exchanging them with an equal volume of TSB solution at the same pH to ensure that total volume remains constant. The released amounts of carvacrol in the different pH conditions were determined by measuring the absorption intensity at a wavelength of 275 nm, using a UV–visible spectrophotometer (SAFAS, Monaco).

To calculate the amounts of released carvacrol from the microcapsules, previously generated standard calibration curves for free carvacrol at different pH were used:

Absorbance = 0.2009*x* + 0.0683, R^2^ = 0.9996; Absorbance = 0.2307*x* + 0.0412, R^2^ = 0.9991; Absorbance = 0.2241*x* + 0.0576, R^2^ = 0.9989; Absorbance = 0.2944*x* + 0.0385, R^2^ = 0.9998; Absorbance = 0.2615*x* + 0.0498, R^2^ = 0.9995; for pH 3.0, 4.0, 5.0, 6.0, and 7.0, respectively. The tests were performed three times, and the cumulative release percentages of carvacrol were obtained using the following equation:

$$Cumulative\;release\;(\%)=\sum_{t=0}^{t}\left(\frac{Q_{t}}{Q_{0}}\right)\times 100$$

where *Q_t_* is the cumulative amount of carvacrol released at each sampling time *t*, and *Q*_0_ is the initial amount of carvacrol loaded in the samples.

### 4.7. Preparation of Different States of Staphylococcus aureus Planktonic Cells, Biofilm, and Biofilm-Detached Cells

Planktonic bacterial suspensions of 10^7^ CFU mL^−1^ were prepared using ¼ Ringer’s solution. For biofilm formation, 5 mL of bacterial suspension (10^6^ CFU mL^−1^) was deposited onto previously sterilized stainless-steel coupons (3 × 1 × 0.1 cm, type AISI-304, Halyvourgiki Inc., Athens, Greece) and incubated for 3 h at 15 °C to promote bacterial cell adhesion to surfaces [87]. After this period, the 5 mL was discarded and the slides were thoroughly double washed with ¼ Ringer’s solution to eradicate non-adhering cells. Next, all slides containing adherent cells were covered with 5 mL of TSB medium and incubated for 48 h at 37 °C by renewing the growth medium every 24 h. Once incubated, the TSB medium was thrown away and the biofilms were double rinsed with ¼ Ringer’s solution to discard planktonic cells. Then, rinsed slides were used to perform antibiofilm treatments and quantify biofilm biomass.

For biofilm-detached cells, after biofilm formation (same protocol), the culture medium was discarded and the biofilms were double washed with ¼ Ringer solution to eliminate weakly attached cells. Then, the biofilm cells were mechanically detached by scraping using cell scraper and harvested in 10 mL of ¼ Ringer solution. Harvested cells were pelleted by centrifugation (5000× *g*, 5 min, 20 °C) and the supernatant was discarded. The pellet was resuspended in 20 mL of ¼ Ringer solution and centrifuged twice. Then, the cells were recovered in 20 mL of ¼ Ringer solution and sonicated at 37 kHz for 5 min at 20 °C to completely remove the EPS matrix [88]. Bacterial suspensions were resuspended in ¼ Ringer’s solution and adjusted to a cell concentration of 10^7^ CFU mL^−1^ to perform the treatment.

### 4.8. Staphylococcus aureus Planktonic Cells, Biofilm and Biofilm-Detached Cells Treatment Using Low pH and/or Microencapsulated Carvacrol

The three different states of bacteria were exposed to the following treatments: (1) different pH ranges of HCl solutions (pH 3, pH 4, pH 5, pH 6, pH 7), (2) microencapsulated carvacrol at the MIC concentration, and (3) combined treatment using microencapsulated carvacrol prepared in HCl solutions at different pH ranges (pH 3, pH 4, pH 5, pH 6, pH 7). Briefly, for planktonic cell and biofilm-detached cell treatments, 0.5 mL of the suspension was added to 4.5 mL of the antimicrobial solutions to obtain a final concentration of 10^6^ CFU mL^−1^, then incubated for 15 min at 15 °C. For biofilm treatment, the prepared biofilms were covered by 5 mL of the antimicrobial solutions and incubated for 15 min at 15 °C. Then, slides were placed into 6 mL of ¼ Ringer solution using a plastic centrifuge tube containing 10 sterile 3 mm diameter glass beads. Biofilm cells were detached by vortexing the plastic tube for 2 min at maximum speed. Suspension of each bacterial state treated with ¼ Ringer solution was used as a control for HCl treatment and suspensions treated with microencapsulated carvacrol prepared in ¼ Ringer solution were used as a control for combined treatment using microencapsulated carvacrol and HCl. Finally, serial dilutions of each bacterial state were prepared in ¼ Ringer’s solution and plated on Tryptic Soy Agar (TSA). Cell numbers were counted on the plates after 24 h incubation at 37 °C and results were expressed

### 4.9. Statistical Analysis

All experiments were repeated twice. The statistical significance was assessed by GraphPad Prism 9.0 software using one-way ANOVA (Dunnett’s multiple comparisons test). *p*-values < 0.05 were defined as statistically significant.

## 5. Conclusions

The results of this study demonstrated that *S. aureus* biofilms exhibited greater resistance to the antimicrobial effects of microencapsulated carvacrol compared to planktonic cells and biofilm-detached cells. This is consistent with extensive prior research showing biofilm-associated bacteria can display 10- to 1000-fold higher antimicrobial resistance than their planktonic counterparts. However, this study also found that the combination of microencapsulated carvacrol and low-pH conditions (pH 3 and pH 4) resulted in a significant reduction in biofilm of over 5 log CFU mL^−1^. This suggests the low pH enhances the antibiofilm activity of the microencapsulated carvacrol. These findings indicate that microencapsulated carvacrol in acidic media could represent a promising alternative approach for removing biofilms from abiotic surfaces in industries like food and healthcare, where conventional antimicrobials have limitations. Further research is warranted to optimize the development of carvacrol-containing acidic microcapsules specifically for combating biofilms. Additionally, continued investigations are needed to identify the most suitable wall materials for encapsulation to improve the efficiency and stability of the microcapsules over time. Future studies should also evaluate the activities of essential oils, either in combination or encapsulated with other antimicrobial compounds, against diverse microbial states beyond just biofilms, such as viable but non-culturable cells, molds, and spores, as well as their impacts on gene expression.

## Data Availability

The authors confirm that the data supporting the findings of this study are available within the article.
